# Round the Hospitals

**Published:** 1918-10-12

**Authors:** 


### ROUND THE HOSPITALS.

Much regret will be felt by all her numerous English friends at the news that Miss Carrie M. Hall, the able Chief Nurse of the American Red Cross, has been transferred to Paris, where she is to hold a similar office to that she has so well discharged here. Her successor in London is Miss Rachel Torrance, of Gowanda, N.Y., who has been working already for four years in different

**ROUND THE HOSPITALS**—(*continued*).

countries under the American Red Cross. She was in Russia with the first American mission during the dreadful 1914 campaign, and later in Rumania. She has not been many months in this country, but has already secured the confidence and affection of her fellow-workers. Her high qualifications and previous record are an earnest that the American Red Cross Nursing Service will continue to prosper under her rule.

The sisters and nurses of St. Thomas's Hospital are to be congratulated on the reputation they have earned as liberal supporters of the War Loans. The Mayor of Lambeth has been so impressed with this that he has made arrangements whereby A Great Howitzer will visit St. Thomas's Hospital next Monday to receive the contributions of the nursing staff to the War Loan, and so help to swell the special effort made to secure that the Howitzers of Trafalgar Square shall produce a bumper for the Chancellor of the Exchequer. We should be glad to hear what other hospitals are doing in this matter, and which hospitals, if any, have also earned the honour of a visit from the Big Gun of War Loan Importance.

A very special interest attaches to the exhibits of the women's section of the Imperial War Museum now on view at the Whitechapel Art Gallery. The most notable feature is Sir George Frampton's bust of Edith Cavell, lent for the occasion, a work of art inspired by rare sympathy and insight. Mestrovic's head of Dr. Elsie Inglis is a fine contrast. The two British women, inspired by the same ideals and both loving not their own lives to the death have proved a brilliant touchstone to two very different types of artist. The model of a bombed hospital, wherein one of the sisters watches by the side of the matron, struck dead at her post, is beyond measure impressive. The specimens of provisional hospital supplies from war depôts will come as a revelation to many.

The name of Miss Constance Watney, chief nurse, C.M.S. Missionary Hospital, Numirambe, Uganda Protectorate, occurs as apparently the only member of the nursing profession in a long list of appointments to the Most Excellent Order of the British Empire for services in or for the Overseas Dominions, Colonies, and Protectorates in connection with the war. The appointments, which were conferred on October 4, date from June 3, 1918, and comprise many well-known people in various parts of the world who have devoted themselves to recruiting, hospital supply, the provision of convalescent homes for the disabled, and other essential duties.

We regret to hear that Miss Carruthers, A.R.R.C., the matron of the Pavilion Hospital, Brighton, has had a very unpleasant accident. She slipped up in the nursing home of the hospital and fell and fractured her thigh. This must be a long and troublesome business, and Miss Carruthers has our heartfelt sympathy in her misfortune.

The retirement of Miss Stansfield after twenty-one years' service from the office of chief lady inspector of the Local Government Board marks an epoch. On her appointment the duties of lady inspectors were ill-defined; their policy was all to make; their subordinates were all to train. With rare insight and immense practical ability she set to work to clear the ground, study the conditions of life among the poor to whom she ministered, and inspire her fellow-workers. The leading features in her policy may be summed up as justice and love. None who have studied the truly educative reports she issued year by year on matters within her department can fail to have been struck by her breadth of mind and literary ability. At a meeting held on September 26 of the Poor-Law Infirmary Matrons' Association the members passed a resolution of sincere regret at her retirement. They put on record their deep gratitude and appreciation of her continued kindness to and consideration for them, not only as matrons in their individual training-schools, but also as members of their association. From its earliest start Miss Stansfeld did all she could to strengthen and encourage the association, which owes much to her support.

The Royal Free Hospital is making radical alterations in its nurse-training school which are greatly to the credit of the authorities. Chief in importance must be mentioned the growth of the maternity department. This has now, owing to the new midwifery school shortly to be opened, assumed dimensions which enable the matron to include training for the C.M.B. examination and certificate in the four years' curriculum. Vacancies for probationers have occurred owing to the new extensions, and the prospect of obtaining a midwifery certificate as part of their normal training will doubtless attract a high class of women. First-year probationers are from now on to receive £16 in lieu of £9. In their second year they advance to £18 instead of £12. All members of the working staff are to have one half-day off duty once each week—a concession which will reduce the average working hours, it is stated, to about eight a day.

A conference of the chairmen of metropolitan hospitals has been suggested by the authorities of the Charing Cross Hospital to discuss the whole question of emoluments, holidays, and hours of work for nurses. Nearly all hospitals have made additions to the salaries of their nursing staffs during the last four years, and at Charing Cross it is announced they will be raised 25 per cent. What is this in money? But all these alterations have been made as it were haphazard, and no fixed principle has emerged. This defect is in a fair way to be removed by the action taken by the College of Nursing. The really important matter, so far as salaries are concerned,

**ROUND THE HOSPITALS—(*continued*).**

is to open up a better career to nurses, not to bribe probationers by high initial payments to enter the profession. The free instruction and enormous advantages open to unskilled women in their early stages of training, at the best schools, do in effect constitute a good salary. It is in the later stage of their institution career, as staff nurses, ward sisters—yes, and as matrons, too—that they are so often shamelessly underpaid.

---

BEST congratulations to Nottingham General Hospital on the appointment of Miss Marie Vaughan Winters as teaching sister. Miss Kendall, whose appointment as matron we chronicled six months ago, has made a notable advance in securing a sister-tutor for her training-school. We believe it to be the first appointment of the kind made in a provincial hospital unattached to a medical school, and it thus marks another milestone on the road to sound professional education. Miss Winters, who was trained at the Royal Infirmary, Leicester, has had wide experience since. Her duties will comprise the theoretical and practical training of all the nurses, with attendance at all lectures given by the hon. medical staff in order to hold classes on them for her pupils.

---

THE inauguration of "one day in seven" for the nurses in the Royal Aberdeen Hospital for Sick Children is an event of first importance for the future of nursing. It is not merely the first hospital in Scotland, it is the first special hospital in the kingdom to bestow this privilege on the staff. In no other branch of nursing is the need for freshness and elasticity, for good spirits, in short, so evident as in the care of sick children. We believe that these qualities are curative in their effects no less than cheering, and that among the best remedies the children can have are the spontaneous little attentions which only nurses unworried by over-fatigue and unhurried in the discharge of their duties can render. Hospital managers may come in time to recognise how swiftly the bestowal of a weekly day of rest to the nurse in charge of little children reacts in favour of the patients. Meantime, in many of our municipal hospitals there are not sufficient nurses in the children's wards to feed the infants by hand, much less to secure the off-duty time required to keep the nurses fresh.

---

THE Irish Branch of the College of Nursing has taken an important step at Dublin towards consolidating its members. A reading- and rest-room (open on week-days from ten to six) has been opened at 23 Kildare Street, Dublin, the offices of the Irish Board. There is a small but good library of professional and other books, and tea is served at a small charge. It meets a distinct need in Dublin, and should develop rapidly into a club of more imposing dimensions.

ABERDEEN has the place of honour this week in the chronicle of progress achieved by the College of Nursing. A Centre of the College was formed last May in this town, of which Miss Edmondson, R.R.C., matron of the Royal Infirmary, Aberdeen, is local representative. The honorary treasurer is Miss Hill, lady superintendent of the Royal Hospital for Children, and the Executive Committee comprises Miss Abel, matron of the Northern Nursing Home; Miss Frater, matron of the City Fever Hospital; Miss MacMaster, superintendent of the District Nursing Association, Q.V.J.I.N.; Miss Munro, matron of the Morningfield Incurables' Hospital; Miss Scott, matron of the Central Nursing Home; and Miss Will, matron of the Aberdeen Poor-Law Hospitals. The names are a token that every type of nurse—general, private, district, children, fever, incurables, Poor-Law—are united in the movement for the higher development of the nursing profession.

---

The object of the meeting of the Aberdeen Centre, held on September 27 in the Royal Infirmary, was to enlist the sympathy and practical support of Aberdeen people in the Nation's Fund for Nurses. The chair was taken by Lady Adam Smith in the unavoidable absence of the Marchioness of Aberdeen. She was able to announce the receipt of £100 from an anonymous donor, a forerunner, it is hoped, of many liberal gifts. Lady Cowdray spoke eloquently on behalf of the cause she has made her own. She placed the need for funds to promote the higher education of nurses in the forefront of her appeal, and enchanted the meeting by the announcement that she would give a nursing scholarship of £100 for competition in Aberdeen on the lines recently followed by the College of Nursing in London.

---

MRS. ALDERTON, member of the Colchester Burgh Council and Education Committee, gave a thoughtful address, emphasising the importance of wider training to nurses in general and their need for club-houses, which in every town in the kingdom should afford them rest, recreation, and opportunities for self-improvement. She pleaded with warmth the cause of the indigent nurse, and begged the Aberdeen people to take up this work seriously. Miss Elizabeth Edmondson, who was greeted with applause, spoke earnestly on the existing need among nurses of co-ordination, affiliation, and registration. She caused some surprise and much satisfaction by urging that nurses ought to have one day's rest in seven secured to them. A capital course of post-graduate lectures and social meetings has been arranged by the Executive Committee of the Aberdeen Centre for this winter, and all marches well.

---

DUNDEE is shortly to form a Centre of the College. On September 26 a meeting was held at Mather's Hotel, Dundee, to explain the objects and organisation of the Nation's Fund for Nurses, at which Lady Cowdray, Lady Baxter, Miss Pegg, matron of the Dundee Royal Infirmary, and others addressed

**ROUND THE HOSPITALS**—*(continued)*.

a keen and sympathetic audience of nurses and the general public. The chairman and hostess was Mrs. Mill. Miss Pegg gave a lucid account of the existing defects in the nurse's training, and of all that the College of Nursing stood for in the eyes of nurses. She also stated clearly the object of the Nation's Fund. Lady Cowdray followed and urged the new educational aims of the profession in a speech which showed her to be remarkably well informed, and an able exponent of the cause. Mrs. Alderton and Mrs. Kinnear also spoke enforcing the need for affiliation and co-operation in the nursing profession. The result of the meeting was to set stirring a strong impulse among Dundee nurses to possess their own Centre of the College.

---

Members of the College will be interested to hear that the annual meeting and conference next year is to take place at Manchester, a cordial invitation having been received from the Manchester Centre to that effect, which the Council have gladly accepted.

---

As we go to press we learn that Miss Gertrude Cowlin, who was appointed temporary organising secretary some months ago, has been formally confirmed in this office by the Council. Miss Rundle remains the chief executive officer of the College. Miss Millicent Ashdown, who temporarily took over Miss Cowlin's duties as Registrar, has now been definitely appointed Registrar of the College. She is the author of "A Complete System of Nursing," and is also examiner in practical nursing at Guy's Hospital and at the West London Hospital.

---

The impending retirement of Miss Heather-Bigg from the matronship of the Charing Cross Hospital, which she has held for over sixteen years, will be learned with regret by a very large circle, lay and professional. The limitations of space have rendered the development of the training-school a task of peculiar difficulty, but Miss Heather-Bigg has never failed to attract a high class of probationer, and her consideration for their comfort and unfailing resourcefulness in dealing with the problems of their cramped surroundings have made their lot a happy one. It has been her custom to welcome probationers who have already received some training in fever, consumptive, children's, eye, or other special hospitals. The training is for four years, and includes in the last year four months' experience in housekeeping for those who desire it, with four months' in electrical and $x$-ray work.

---

In the course of her term of office Miss Heather-Bigg has seen the hospital pass through troubled times. Her wide experience and energy have assisted immeasurably in the developments of the last year or two, during which, as was recently noted in The Hospital, a new chapel, theatre, and mortuary have been built or opened, new nurses' quarters have been provided for part of the staff, the kitchens, heating, and lighting have been modernised, a tuberculosis dispensary, a child welfare clinic, and a clinic for venereal disease have been opened.

---

Poor-Law infirmaries are by no means to be classed in one category in respect of their children's wards. We have recently visited the Whipps Cross War Hospital, or West Ham Infirmary, where, in spite of the claims of 600 wounded men, the matron, Miss Clark, keeps up a high standard of nursing among the sick poor. In the children's wards the average is four beds to each member of the day and night staff, eight by day and eight by night. Below this lies danger. That it is possible with this average to keep the wards cheerful and the children well cared for is exemplified at Whipps Cross, where every baby expects notice and claims a finger to clutch as the sister passes by, where softly-padded "playgrounds" near the fire are filled with jolly two-year-olds, and where vacancy, mechanical movements, lifeless eyes, are conspicuously absent. Miss Clark, as is well known, has been promised one day in seven for her nurses by the West Ham Guardians when the war is over.

---

Considerable difficulty and some friction has arisen in connection with the Nurses' Home at the Staincliffe Poor-Law Institution, Dewsbury. The home contains between sixty and seventy nurses, and the entire staff, superintendent nurse, sisters, and nurses, are agreed in desiring the appointment of a home sister instead of a lay housekeeper. The house committee have convinced themselves that a trained nurse set over the home would have a better understanding of the nurses' difficulties and more sympathy with them in the strain involved by remaining "on duty, as often happened, for twelve or more hours at a stretch." Though warmly supported by many present, the proposal to appoint a home sister was referred back for inquiry to be made as to the practice at similar institutions—a singularly weak decision, for why, in any case, should not Dewsbury lead the way? The Guardians would be wise to inquire at the same time whether there be any young woman capable, without injury to health, of remaining on duty *frequently* for twelve or more hours at a stretch.

---

The Hammersmith Public Health Committee are trying to put pressure on the Government to secure a proper and sufficient supply of milk. A very grave increase in cases of adulteration has come to light, and we believe many hospitals have had to register complaints concerning the quality of the milk supplied at the present time. It is satisfactory to learn that the Food Controller is taking control of the wholesale milk supply. Meantime, extra precautions should be taken by all who have to do with the reception and storage of milk in institutions. Undoubtedly the most vigilant customers are the best served.

